# Efficacy of adding the kinesio taping method to guideline-endorsed conventional physiotherapy in patients with chronic nonspecific low back pain: a randomised controlled trial

**DOI:** 10.1186/1471-2474-14-301

**Published:** 2013-10-24

**Authors:** Marco Aurélio Nemitalla Added, Leonardo Oliveira Pena Costa, Thiago Yukio Fukuda, Diego Galace de Freitas, Evelyn Cassia Salomão, Renan Lima Monteiro, Lucíola da Cunha Menezes Costa

**Affiliations:** 1 Physical Therapy Department, Santa Casa Misericórdia de São Paulo, Brazil, Rua Dr Cesário Motta Jr, 112, 01221-020 São Paulo-SP, Brazil; 2 Masters and Doctoral Programs in Physical Therapy, Universidade Cidade de São Paulo, Rua Cesário Galeno 475, 03071-000 São Paulo-SP, Brazil; 3 Musculoskeletal Division, The George Institute for Global Health, Sydney, NSW, Australia

## Abstract

**Background:**

Chronic nonspecific low back pain is a significant health condition with high prevalence worldwide and it is associated with enormous costs to society. Clinical practice guidelines show that many interventions are available to treat patients with chronic low back pain, but the vast majority of these interventions have a modest effect in reducing pain and disability. An intervention that has been widespread in recent years is the use of elastic bandages called Kinesio Taping. Although Kinesio Taping has been used extensively in clinical practice, current evidence does not support the use of this intervention; however these conclusions are based on a small number of underpowered studies. Therefore, questions remain about the effectiveness of the Kinesio Taping method as an additional treatment to interventions, such as conventional physiotherapy, that have already been recommended by the current clinical practice guidelines in robust and high-quality randomised controlled trials. We aim to determine the effectiveness of the addition of the use of Kinesio Taping in patients with chronic nonspecific low back pain who receive guideline-endorsed conventional physiotherapy.

**Methods/design:**

One hundred and forty-eight patients will be randomly allocated to receive either conventional physiotherapy, which consists of a combination of manual therapy techniques, general exercises, and specific stabilisation exercises (Guideline-Endorsed Conventional Physiotherapy Group) or to receive conventional physiotherapy with the addition of Kinesio Taping to the lumbar spine (Conventional Physiotherapy plus Kinesio Taping Group) over a period of 5 weeks (10 sessions of treatment). Clinical outcomes (pain intensity, disability and global perceived effect) will be collected at baseline and at 5 weeks, 3 months, and 6 months after randomisation. We will also collect satisfaction with care and adverse effects after treatment. Data will be collected by a blinded assessor. All statistical analysis will be conducted following the principles of intention to treat, and the effects of treatment will be calculated using Linear Mixed Models.

**Discussion:**

The results of this study will provide new information about the usefulness of Kinesio Taping as an additional component of a guideline-endorsed physiotherapy program in patients with chronic nonspecific low back pain.

## Background

Low back pain is a significant public health condition and it is associated with a high rate of absenteeism from work, disability, and frequent use of health services [1]. Approximately 39% of the population suffers from low back pain at some stage in their lives [2,3]. The Brazilian National Survey by Household Sample (PNAD, 2010) [4] ranked back pain as the second most prevalent health condition after systemic arterial hypertension [4]. This high prevalence explains the vast amounts expended on treatment for patients with this condition. The most recent systematic review on the cost associated with low back pain indicates that the majority of direct costs were spent on physiotherapy (17%), followed by medication (13%) and other primary health care (13%), however these costs account for less than 20% of the total costs of this condition, i.e. most of the costs are related to indirect expenses with absenteeism from work and lower productivity [5].

Current literature provides several possibilities for the treatment of low back pain that vary according to duration of symptoms and classification of this condition [6,7]. These treatments range from educational programs [8] to behavioural cognitive therapy [9], medication [10], electrophysical agents [11], manual therapy [12-14] (e.g. joint mobilisation/manipulation, myofascial release), general exercises [15] and specific spinal stabilisation exercises [16], among others [7]. Although clinical practice guidelines recommend the aforementioned treatments for patients with chronic nonspecific low back pain, most randomised controlled trials, from which the guidelines are taken, have shown that these treatments provide only mild to moderate clinical improvement in these patients when used in isolation [7,12,16]. These same clinical practice guidelines also state that there is no difference between the various modalities of exercise-based therapy as well as the various manual therapy techniques [7].

Given the modest clinical improvement and the lack of a leading therapy, new interventions are being tested within the variety of physiotherapy techniques to enhance the effect size of the treatment being used and thus increase patient satisfaction. A new treatment option that is very popular in athletes is the Kinesio Taping and it is being widely used in patients with low back pain. This method was created in Japan by Kenso Kase in the 70's [17]. The technique uses an elastic tape that is extremely thin and much more elastic than conventional bandages and applies it to the patient’s skin. This tape can be stretched to 140% of its original length, producing less mechanical retention and restriction to movement [17]. During assessment, the therapist decides which technique and level of traction to give the bandage, generating more or less tension on the skin. According to its developers, this traction elevates the epidermis increasing the pressure on the mechanoreceptors below the dermis, thus decreasing nociceptive stimuli. The creators of the Kinesio Taping also state that the tape is able to improve blood and lymphatic circulation, reduces pain, realigns joints, and reduces muscle tension [17,18]. Additionally, the use of Kinesio Taping is likely to change the pattern of recruitment of muscle fibres [18-20]. In the case of the latter, which involves great activation of the paravertebral musculature in response to pain, it is expected that the use of bandages (such as Kinesio Taping) would inhibit this excessive activation, thus increasing range of motion and, subsequently, will improve functionality and would reduce pain intensity [19-21].

There are three systematic reviews on the use of the Kinesio Taping in patients with musculoskeletal conditions [22-24]. All reviews were consistent in concluding that there is no high-quality evidence of the use of Kinesio Taping in patients with musculoskeletal conditions, including patients with chronic low back pain. However, most of the clinical trials used Kinesio Taping in isolation, had small samples, and had high risk of bias. From a pragmatic standpoint, Kinesio Taping is not used by physiotherapists as an isolated form of intervention, but as an additional component in the treatment of patients with low back pain in order to increase and prolong the effect of pain reduction and disability in these patients.

Given that most patients with chronic nonspecific low back pain receive a variety of interventions within the scope of conventional physiotherapy (advice/counselling, manual therapy techniques, general exercise, and specific spinal stabilisation exercises), the present study intends to investigate whether the addition of Kinesio Taping to conventional physiotherapy treatment can provide greater pain relief and functionality than conventional physiotherapy alone in patients with chronic nonspecific low back pain.

### Objective

The primary objective of this trial protocol will be to investigate the efficacy of the addition of the use of Kinesio Taping in relieving pain and improving disability in patients with chronic nonspecific low back pain treated according to the principles of conventional physiotherapy (based on the clinical practice guidelines) compared to patients treated only with conventional physiotherapy. An assessment will be conducted immediately after the treatment (5 weeks after randomisation) (primary outcomes).

The secondary objectives of this study will be:

To analyse the difference between the group that will receive Kinesio Taping in addition to conventional physiotherapy treatment and the group that will be treated only with conventional physiotherapy in the outcomes pain intensity (pain relief) and disability assessed 3 and 6 months after randomisation (secondary outcomes).

To analyse the difference between the group that will receive Kinesio Taping in addition to conventional physiotherapy treatment and the group that will be treated only with conventional physiotherapy in the outcome global perceived effect assessed 5 weeks, 3 months, and 6 months after randomisation (secondary outcomes).

To analyse the patient’s adherence to and satisfaction with the treatment (secondary outcomes).

### Hypothesis

The hypothesis of this study is that the patients with chronic nonspecific low back pain who receive conventional physiotherapy treatment in addition to Kinesio Taping will have greater reduction in pain intensity levels, better global perceived effect, and less disability compared to patients who receive only conventional physiotherapy treatment as assessed immediately after the 5 week intervention and that these benefits will be maintained until the reassessments 3 and 6 months after randomisation.

## Methods/Design

### Study design

This study will be a two-arm randomised controlled trial, prospectively registered, and with blinded assessor.

### Approval and registration

The procedures and consent form were approved by the Research Ethics Committee of Universidade Cidade de São Paulo (protocol no. 254.063), and the study is being fully funded by Fundação de Amparo à Pesquisa do Estado de São Paulo (FAPESP) (2013/02075-8). The study will be conducted at the outpatient physiotherapy clinic of Irmandade da Santa Casa de Misericórdia de São Paulo, in São Paulo, Brazil. This study was prospectively registered at ClinicalTrials.gov - NCT01866332.

### Sample size calculation

The sample size calculation for this study was based on the detection of a one-point difference between groups for the outcome pain intensity assessed by the Pain Numerical Rating Scale [25] (estimated standard deviation of 1.84) and a four-point difference for the outcome disability measured by the Roland Morris Disability Questionnaire [25,26] (estimated standard deviation of 4.9 points) with a statistical power of 80%, alpha of 5%, and possible sample loss of up to 15% [16]. Therefore, 74 participants were needed per group or 148 in total.

### Participants

We will recruit participants of both genders between 18 and 60 years of age with chronic nonspecific low back pain for more than three months and who are seeking treatment for low back pain. Participants will be excluded if they have any contraindications to physical exercise according to the guidelines of the American College of Sports Medicine [27]; serious spinal pathologies (fractures, tumors, and inflammatory pathologies such as ankylosing spondylitis); nerve root compromise (disc herniation and spondylolisthesis with neurological compromise, spinal stenosis, and others); contraindications to the use of Kinesio Taping (allergy or intolerance), serious cardiorespiratory diseases or pregnancy.

### Assessment procedures

The potential study participants will be referred to their respective medical doctors for all assessments and routine examinations, such as imaging tests, prescription of medication, and referral for physiotherapy. The participants will be sent to the physiotherapy clinic where they will be assessed and included or excluded from the study according to the aforementioned eligibility criteria. They will be informed about the study’s objectives, timeline, and eligibility criteria, then asked to sign an informed consent form if they agree to take part in the study.

If the participant is considered eligible, the assessor will collect the baseline data prior to randomisation. This assessor will be blinded to patient allocation to treatment groups. The following instruments will be used to assess the participants: 1) Assessment Form; 2) Pain Numerical Rating Scale [25]; 3) Roland Morris Disability Questionnaire [25,26]; 4) Global Perceived Effect Scale [25]; and 4) MedRisk Instrument for Measuring Patient Satisfaction With Physical Therapy Care [28,29]. All scales and questionnaires have been translated and cross-culturally adapted to the Brazilian population, and their respective measurement properties have been assessed by our research group [25,29,30]. A detailed description of each of the instruments is given below.

### Assessment instruments

#### Assessment form

Participant characteristics will be collected with the use of an assessment form designed specifically for this study. This form will contain questions regarding demographic and anthropometric data, as well as the participant’s health condition, such as use of medication, level of physical activity, educational level, history of low back pain and duration of symptoms.

#### Pain numerical rating scale

The Pain Numerical rating Scale assesses the pain intensity levels perceived by the patient using an 11-point scale (ranging from 0 to 10), with 0 representing “no pain” and 10 representing “the worst possible pain”. The participants will be instructed to report the level of pain intensity in the last seven days [25].

#### Roland Morris disability questionnaire

The Roland Morris Disability Questionnaire assess disability associated with low back pain by means of 24 questions that describe daily tasks that the patients have difficulty performing due to low back pain [25,26]. The patients will be instructed to answer the questions that actually apply to them over the last 24 hours. The total score is the sum of the points obtained, ranging from 0 to 24 points. The higher the number of answers is, the higher the disability.

#### Global perceived effect scale

The Global Perceived Effect Scale assessed the global impression of recovery as perceived by the participant comparing the onset of symptoms to the last few days. It is an 11-point numerical scale ranging from -5 (vastly worse) to 0 (unchanged) to +5 (completely recovered). To measure the global impression of recovery, the participants will be asked: “Compared to when this episode first started, how would you describe your back these days?”. Higher scores indicate better recovery [25,31].

#### MedRisk instrument for measuring patient satisfaction with physiotherapy care

MedRisk is an instrument used to assess the satisfaction of patients who receive physiotherapy care. It is composed of 20 items, including 10 items related to physiotherapist-patient interaction, such as “My therapist answers all of my questions” (item 14); 8 items are not related to physiotherapist-patient interaction, e.g. the office receptionist’s courtesy (item 1); and, finally, 2 items that are considered global items, such as “I would return to this clinic for future services” (item 20). The patients will select their level of satisfaction for each item on a Likert-type scale that varies from 1 (strongly disagree) to 5 (strongly agree) or use the option “not applicable”, with high scores representing high satisfaction [28,29].

All of these assessment instruments will be collected at baseline and 5 weeks, 3 months, and 6 months after randomisation, except for the assessment form, which will be completed only at baseline, and the MedRisk instrument, which will be applied during the 5-week assessment to describe satisfaction with the treatment received. We will also monitor any adverse events that could happen over the treatment period, such as exarcebation of pain, allergy and others.

### Primary outcomes

1 Pain intensity perceived by the participant measured by the Pain Numerical Rating Scale at 5 weeks after randomisation;

2 Disability measured by the Roland Morris Disability Questionnaire at 5 weeks after randomisation;

### Secondary Outcomes

1 Pain intensity perceived by the participant measured by the Pain Numerical Rating Scale [25] at 3 months and 6 months after randomisation;

2 Disability measured by the Roland Morris Disability Questionnaire [25,26] at 3 and 6 after randomisation;

3 Global impression of recovery measured by the Global Perceived Effect Scale [25] at 5 weeks, 3 and 6 months after randomisation.

### Other outcomes

Patient satisfaction with physiotherapy care measured by the MedRisk scale [28,29] at 5 weeks after randomisation.

Adverse events will be monitores over the course of treatment and at 5 weeks after randomisation.

The assessor who will collect the data related to the assessment instruments in every assessment of the study (baseline, 5 weeks, 3 months, and 6 months after randomisation) will not be aware of the treatment the participants will receive.

### Allergy test

All participants considered eligible for the study will undergo a Kinesio tape allergy test immediately after the initial assessment (but before randomisation). This test consists of sticking a small piece of Kinesio tape to the thoracic spine and leaving it for 24 hours. The patients who develop an allergic reaction to the tape will be asked to remove it immediately and will not be included in the study. After this allergic test, the allergy-free patients will be randomised to the treatment groups.

### Random allocation of patients

**Figure 1:**
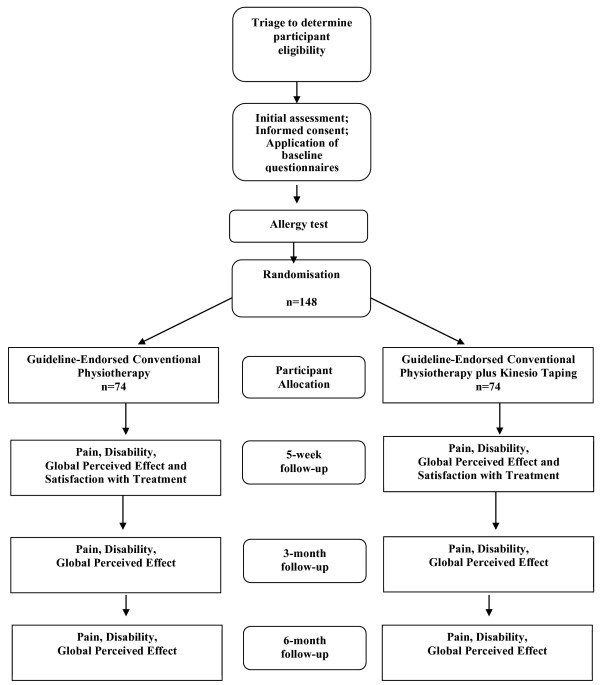
Study flow diagram.

Immediately after the initial assessment and the allergy test, the participants will be referred to the therapist overseeing the treatment. Before the start of treatment, the participants will be randomly allocated to two groups: Guideline-Endorsed Conventional Physiotherapy Group submitted to manual therapy techniques, general exercise, and specific spinal stabilisation exercises or Guideline-Endorsed Conventional Physiotherapy Group plus Kinesio Taping submitted to the same treatment as the previous group plus Kinesio Taping. Allocation will be conducted according to a computer-generated randomisation schedule performed by a researcher not involved in participant recruitment, assessment or treatment. Participant allocation will be concealed using a random numerical sequence in sealed opaque envelopes. Before beginning the intervention, the therapist overseeing treatment will open the envelope in front of the patient and will disclose the treatment technique that corresponds to the number in the envelope. Figure 1 provides a visual reference of the study design.

### Interventions

In this study, 148 participants will be randomly allocated to receive 10 treatment sessions of conventional physiotherapy, consisting of manual therapy techniques, general exercise, and specific spinal stabilisation exercises (Guideline-Endorsed Conventional Physiotherapy Group) or application of Kinesio Taping to the lumbar spine in addition to the aforementioned treatment (Guideline-Endorsed Conventional Physiotherapy plus Kinesio Taping Group). Sessions will last 30 to 60 minutes and will be held twice a week for 5 weeks, for a total of 10 sessions. Before the start of the treatment period, the participants will receive basic orientation regarding the methods that will be used.

The participants allocated to the Guideline-Endorsed Conventional Physiotherapy Group will receive the following treatment: 1) manual therapy techniques consisting of joint mobilisation using the Maitland approach [32], in which the posteroanterior central (PAC) pressure technique will be applied in three series of one minute each (1-minute interval between series) to the vertebral segment that is hypomobile or painful; another manual therapy technique that will be used is myofascial release [13,14,33], with manual ischemic compression of the previously assessed band of tension for 30 to 60 seconds. These manual techniques aim to reduce muscle activity and stiffness, improving lumbar range of motion; 2) general exercise aimed at increasing the patients’ level of physical activity (including simple exercises such as short walks, stretching, and strengthening of the major muscle groups, such as gluteus and rectus abdominis) [6,7]; and 3) specific spinal stabilisation exercises consisting of motor control training of the transversus abdominis and multifidus muscles in static and functional activities [16,34-37]. The therapist will teach the patients to contract these muscles by using verbal commands and palpation. Once the participant learns to contract the muscles, the contractions will be combined with exercises following a protocol previously developed by the researchers [16], which will include breathing exercises, active movement of upper and lower limbs, change from supine to prone, and increase in difficulty level according to individual ability to maintain muscle contraction. If a particular exercise is too difficult for the participant (due to sedentarism, weakness or pain), it will be interrupted, and the protocol will continue starting with the previous exercise. The main objective of the specific exercises is to restore the patterns of muscle contraction, improve movement of spinal muscles, and increase joint protection through muscle contraction.

**Figure 2:**
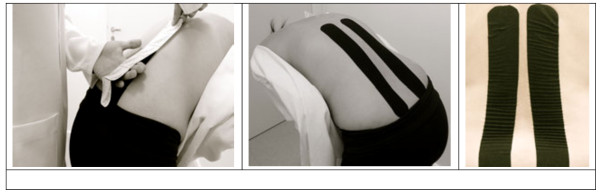
**Application of Kinesio Tape **[38]**.**

The participants allocated to the Guideline-Endorsed Conventional Physiotherapy plus Kinesio Taping Group will receive the same treatment as the Guideline-Endorsed Conventional Physiotherapy Group (joint mobilisation, myofascial release, and segment stabilisation) and, at the end of each session, Kinesio Taping will be applied to the lumbar spine. The Kinesio Taping technique uses elastic bandages (5 cm wide and 0.5 mm thick) that are fixed to the skin of the area being treated. These bandages are 100% cotton, breathable, and do not restrict range of motion. The adhesive is heat-activated and latex-free, considerably reducing the risk of allergy or skin reactions. During the manufacturing process, the bandage is fixed to the backing paper at 10-15% tension. Its durability is 3–5 days and it can even be worn in the water as it only expands longitudinally [17]. In this study, the bandage will be positioned on the paravertebral muscles (bilaterally) parallel to the spinous processes of the lumbar spine, starting near the posterior superior iliac spine at the level of the T12. Firstly, the initial anchor point will be applied to the sacral region (at the S1) without tension (0%). After that, the participant will be asked to flex the trunk and the bandage will be applied in the shape of an “I” over the skin in the paravertebral region up to the extremity of the T12 vertebra at 10-15% tension (tension from the backing paper), and finally the final anchor point will be fixed directly above the T12 with 0% tension, according to the principles of the technique (Figure 2) [17]. This technique was used in another clinical trial performed by our research group [38].

Both treatments will be conducted by physiotherapists trained in the methods of joint mobilisation (Maitland), myofascial release, and segment stabilisation and in the Kinesio Taping method. The chief investigator of this study is a certified Kinesio Taping therapist (levels KT 1 and KT 2). These treatments will be applied according to the participant’s clinical status, therefore the exercises and the manual therapy techniques will be individualised according to the clinical examination. This procedure faithfully represents the procedures of physiotherapists in clinical practice.

### Statistical analysis

All statistical procedures will be performed according to the principles of intention to treat [39]. First, descriptive analyses will be conducted to determine data normality (or lack thereof). The between-group comparisons to obtain the mean effects of the treatments will be conducted by means of interaction terms (group versus time interactions) using Linear Mixed Models. The statistical analysis will be conducted by a researcher who will not be involved in any of the phases of data collection and will receive data in coded form and therefore is considered as blinded. The SPSS 19 will be used for these analyses.

## Discussion

This study will investigate a condition that is clinically significant for physiotherapists, and the results will provide reliable information that will guide the future use of the Kinesio Taping method in patients with non-specific low back pain. Regardless of the findings of the present study, the results will be considered important. For example, if adding Kinesio Taping to guideline-endorsed conventional physiotherapy provides greater pain relief and functionality improvement than conventional physiotherapy alone, this method could be confirmed as an effective treatment for these patients. If, on the other hand, the present study does not find any difference between the intervention groups, the role of Kinesio Taping in assisting pain reduction and functionality improvement will have to be reconsidered, especially taking into account the added costs of treating patients with chronic nonspecific low back pain with this increasingly popular method in clinical practice.

## Competing interests

The authors declare that there is no conflict of interest related to this manuscript.

## Authors’ contributions

MANA, LOPC, TYF, DGF, ECS, RLM, LCMC were responsible for the design of the study. LCMC and LOPC procured funding. MANA and LCMC drafted the manuscript and all authors have contributed to the manuscript. All authors have read and approved the final manuscript.

## Pre-publication history

The pre-publication history for this paper can be accessed here:

http://www.biomedcentral.com/1471-2474/14/301/prepub
